# Commentary on Interictal epileptogenic zone localization in patients with focal epilepsy using electric source imaging and directed functional connectivity from low‐density EEG

**DOI:** 10.1002/epi4.12426

**Published:** 2020-08-26

**Authors:** Lara Jehi

**Affiliations:** ^1^ Cleveland Clinic Cleveland OH USA

Resective epilepsy surgery is the most effective treatment option for patients with drug‐resistant focal epilepsy. Accurate localization of the epileptogenic zone (EZ) is necessary to achieve seizure freedom postoperatively. In challenging cases, this localization requires extensive testing, often including intracranial electroencephalography (iEEG) and complex neuroimaging. Multiple postprocessing tools aiming to “optimize the localization value” of such expensive and sophisticated work‐ups have exclusively focused on analyzing iEEG. On the upside, this undivided attention to iEEG has greatly advanced our understanding of epilepsy electrophysiology. On the downside, this lack of intellectual diversification is leaving all other sources of electrophysiological data, including the foundational scalp EEG, woefully under‐exploited. Herein lies the value of DrCoito's paper,^1^ selected for this year's Epilepsia Open Clinical Prize.

DrCoito and her team investigated the accuracy of electrical source imaging (ESI) and directed functional connectivity (DFC) based on the low‐density clinical scalp EEG. ESI is a noninvasive technique that uses mathematical algorithms to localize the source of a given EEG pattern in real time,^2^ while DFC refers to methods that appeal to temporal precedence of electrophysiology or imaging data to construct macroscopic neural networks and infer directed connectivity.^3^ The classical study subject matter for both ESI and DFC has been high‐density EEG or iEEG. Instead, Coito et.al asked how much we can learn using “everyday's” EEG before more invasive and complex procedures are done. The answer? Quite a bit, although as we will see, the strength of any conclusions needs to be tempered by the typical limitations of exploratory work.

The authors selected 20 patients with temporal lobe epilepsy (TLE) and 14 with extratemporal lobe epilepsy (ETLE) who had a focal magnetic resonance imaging lesion, invasively well‐characterized presumed EZ, and ≥10 interictal epileptic discharges of the same (single) type on low‐density EEG. They then estimated the cortical activity during each interictal spike in 82 cortical regions using a patient‐specific head model. The study team computed DFC between brain regions using Granger‐causal modeling followed by network topologic measures. Ultimately, concordance with the presumed EZ at the sublobar level was calculated using the epileptogenic lesion or the resected area in postoperative seizure‐free patients. The findings were encouraging. The percentage of concordant patients for ESI was 76% (90% in TLE and 57% in ETLE, *P* = .026). Similarly, high connectivity was demonstrated, with 76% concordant patients for the summed outflow (95% in TLE and 50% in ETLE, *P* = .002), 70% for the clustering coefficient (85% in TLE and 50% in ETLE, *P* = .027), and 70% for the betweenness centrality (80% in TLE and 57% in ETLE, *P* = .15), and 76% for the efficiency (90% in TLE and 50% in ETLE, *P* = .009). DFC did not outperform ESI. Altogether, both seemed to provide reasonable adjunctive localizing value of high sensitivity based on interictal findings alone. Given that seizures might not always be detected during clinical evaluation, localizing information during interictal spikes is very relevant. Similar encouraging results were seen in the subgroup with normal MRI, albeit a small sample size of six patients.

Strengths of the paper are multiple. This is the first study using low‐density scalp EEG during interictal periods with the aim of validating DFC measures. The patient population is thoroughly described, as well as the EEG and imaging analyses. The use of a realistic head model improved localization with ESI: The head was divided into 6 compartments (the scalp, bone, cerebral spinal fluid, gray matter, white matter, and air) allowing for more sophisticated modeling of the electrophysiology in low‐density scalp EEG. Showing the discrepancy in the localization accuracy between TLE and ETLE highlighted the significant need for better tools in ETLE. A subgroup analysis of the correlations between the number of recording electrodes and EZ estimation explored whether localization yield can be improved by adding electrodes. Several limitations are worth noting too. The study did not include patients with multiple foci limiting the applicability of the findings to this complex patient population most in need of assistance with EZ localization. The study only included operated patients who had a good surgical outcome preventing any assessment of specificity for the ESI or DFC measures. Lastly, the true significance of higher concordance in TLE vs ETLE cannot be judged given the fact that TLE resections were larger than those in ETLE, which by itself increases the likelihood of including an area of abnormal ESI measurements in the resection.

Ultimately, one cannot and should not translate observations of concordance or lack thereof from retrospectively selected cohorts into statements assigning any predictive value in future unbiased patient cohorts. In this situation, for example, patients who may have had a “robust” localization with ESI—but one that was very discordant with other presurgical tests or seizure semiology—were likely not operated upon or had resections that involved other brain regions supported by the rest of their testing. In essence, it is not the resection of the ESI localization per se that made the patient seizure‐free, but rather the fact that this localization was concordant with every other presurgical test facilitating the localization of the epilepsy. Coito et.al know better and call for larger prospective studies to validate their findings. Meanwhile, one can learn more about their worthwhile and interesting findings in this issue.

Read the winning article ‐ https://onlinelibrary.wiley.com/doi/abs/10.1002/epi4.12318

## CONFLICT OF INTEREST

The author has no conflict of interest to disclose. I confirm that I have read the Journal's position on issues involved in ethical publication and affirm that this report is consistent with those guidelines.
